# Toxicity Evaluation of Nanostructured Silica Orally Administered to Rats: Influence on Immune System Function

**DOI:** 10.3390/nano10112126

**Published:** 2020-10-26

**Authors:** Ivan V. Gmoshinski, Vladimir A. Shipelin, Antonina A. Shumakova, Eleonora N. Trushina, Oksana K. Mustafina, Irina V. Safenkova, Sergey A. Khotimchenko, Dmitry B. Nikityuk, Viktor A. Tutelyan

**Affiliations:** 1 Federal Research Centre of Nutrition and Biotechnology, 109240 Moscow, Russia; gmosh@ion.ru (I.V.G.); antonina_sh@list.ru (A.A.S.); trushina@ion.ru (E.N.T.); mustafina@ion.ru (O.K.M.); hotimchenko@ion.ru (S.A.K.); dimitrynik@mail.ru (D.B.N.); tutelyan@ion.ru (V.A.T.); 2 Plekhanov Russian University of Economics, 115093 Moscow, Russia; 3 Research Centre of Biotechnology of the Russian Academy of Sciences, A.N. Bach Institute of Biochemistry, 119071 Moscow, Russia; saf-iri@yandex.ru; 4 I.M. Sechenov First Moscow State Medical University (Sechenov University), 119991 Moscow, Russia

**Keywords:** silica, nanoparticles, rats, subacute toxicity, leucocytes, cellular immunity, cytokines, antibodies, systemic anaphylaxis, NOAEL

## Abstract

The experimental data on the oral toxicity of nanostructured amorphous silica (SiO_2_), widely used in food supplements, pharmaceuticals, and cosmetics, in terms of its in vivo effect on the immune system, are contradictory. Therefore, this study aimed to assess the rat’s immune function after SiO_2_ oral administration. In the first experiment, SiO_2_ was daily orally administered to Wistar rats for 92 days in doses of 0.1, 1.0, 10, and 100 mg/kg of body weight (bw). In the second 28-day experiment, SiO_2_ in a dose of 100 mg/kg bw was daily orally administered to rats parenterally immunized with the food allergen ovalbumin (OVA) for the reproduction of systemic anaphylaxis reaction. Together with integral indices, we assessed intestinal permeability to protein macromolecules; hematology; CD45RA+, CD3+, CD4+, CD8+, and CD161a+ cells; cytokines TNF-α, IL-6, and IL-10; and IgG to OVA. The results obtained showed that SiO_2_ has no effect on the severity of the anaphylactic reaction, but is capable inducing a toxic effect on the T-cell immune systems of rats. Estimated no observed adverse effect level NOAEL for SiO_2_ ranges up to 100 mg/kg bw in terms of its daily consumption for 1–3 months. Using SiO_2_ as a food additive should be the subject of regulation.

## 1. Introduction

Amorphous silicon dioxide (silica, SiO_2_) is widely used nowadays as a food additive (E551), and also as parts of a large number of tableted pharmaceuticals, many types of cosmetic products, and synthetic and natural flavors. The role played by silicon dioxide added to the listed kinds of products is reduced to its function as an anti-caking agent and carrier. In the present specification Joint FAO/WHO Expert Committee on Food Additives (JECFA) for SiO_2_ as a food additive [1], there is no information about the size of its particles, which allows the use of superfine amorphous SiO_2_, obtained by gas-phase hydrolysis of tetrachlorosilane of high purity. This material, also known as “Aerosil^®^”, has the size of the primary nanoparticles (NPs) forming in the reactor of within 6–30 nm. These NPs subsequently form loose aggregates of the submicron size which are prone to dissociation under the influence of mechanical impact. Consequently, this form of SiO_2_ can be considered as consisting basically of NPs, so this is a nanomaterial (NM). Contents of SiO_2_ with particle sizes on the nanoscale in the food, cosmetic, and pharmaceutical products are not currently controlled, wherein the data about the possible toxicity of silica nanoparticles (NPs) with different sizes, porosity, and surface chemistry are contradictive. Toxicity of nanostructured amorphous SiO_2_ at the inhalation route of intake can be significant [2,3], whereas results that have been obtained with its oral route of intake do not coincide with each other. Hence, in particular, even a single administration to rats of monodisperse SiO_2_ NPs results in a series of both local and systemic toxic effects which are evident at a dose of less than 1000 mg/kg of body weight (bw) [4,5]. However, in the study [6] only marginal toxic effects of SiO_2_ nanoparticles manifested at a dose of 2500 mg/kg bw or more with repeated intragastric administration, and another study [7] did not reveal any adverse effects for both nanoparticles and microparticles of amorphous silica.

There is evidence of the ability of silica NPs to exert endothelial inflammation with hemostatic and thrombosis effects [8], and influence the state of the immune system [9,10], in particular, the severity of allergic reactions in the inhalation and intranasal route of entry [11].

The present paper aims to evaluate the influence of orally administered nanostructured SiO_2_ used as a food additive in pharmacy and cosmetics on the immune function of rats in order to establish, on the basis of these data, a dose of this NM not causing adverse effect (NOAEL—no observed adverse effect level).

## 2. Materials and Methods

### 2.1. Characterization of Nanomaterial

Studied ultrafine amorphous SiO_2_ was purchased in company “Silica” LTD (Russia, Moscow district, Dolgoprudny town) under the trademark “Orysil 300”. Index “300” in the nomination of the product means specific surface in m^2^/g measured by the method of adsorption isotherms of inert gases. The product is an X-ray amorphous light white powder, giving upon sonication, opalescent colorless dispersion (colloidal solution) in water stable for at least two days. According to the manufacturer’s data, SiO_2_ particles in the product were not porous. Silica particles were no coated with any organic materials.

Estimation of the size and shape of the particles in the product was made by atomic force microscopy (AFM), transmission electron microscopy (TEM), spectroacoustic method, and dynamic laser light scattering (DLS).

When studied by AFM, the SiO_2_ powder in an amount of 10 mg was suspended in 5 mL deionized H_2_O and sonicated (duration 5 min, frequency 44 kHz, power 1 W/cm^3^). The suspension (5 µL) was placed on the surface of freshly cleaved mica by dipping and incubated for 10 min at room temperature. Then, the excess liquid was removed by filter paper and dried. The AFM was performed using a Smart SPM-1000 atomic force microscope (AIST-NT, Moscow region, Russia) in a tapping mode. For high resolution, we used fpN01HAR cantilevers (Nanotuning, Moscow region, Russia) totally characterized and calibrated by the manufacturer and having a tip radius curvature of about 1 nm. We prepared three identical specimens and measured two times for each sample. The samples were sequentially scanned in areas with the following dimensions: 35 × 35 µm^2^, 20 × 20 µm^2^, 5 × 5 µm^2^, and 1 × 1 µm^2^. The obtained images were analyzed using the Gwiddion software v.2.42 (Czech Metrology Institute, Czech Republic).

TEM study of the sizes and shapes of the particles of SiO_2_ preparation was done by transmission electron microscope JEM-100SKH (JEOL, Japan) at an accelerating voltage of 80 kV. Before the study, a suspension of SiO_2_ with a concentration of 1 µg/cm^3^ in water was processed in the same way as indicated in the case of AFM, sedimented on carbon-coated copper grids, and dried.

Spectroacoustic study of SiO_2_ dispersion was performed using a DT-1202 analyzer (Dispersion Technology Inc., Bedford Hills, NY, USA) in accordance with the method [12].

DLS study of SiO_2_ particles’ distribution by size (hydrodynamic diameter) was made in particle analyzer Nanotrack Wave (Microtrac Inc., York, PA, USA). The data were analyzed with the assumption of the presence of spherical opaque particles using software FLEX 11.0.0.1 (Microtrac Inc., York, PA, USA). In the cases of spectroacoustic and DLS studies, the suspension of SiO_2_ in deionized water was sonicated immediately before the study with duration 5 min, frequency 44 kHz, and power 1 W/cm^3^ on a submersible ultrasonic processor.

### 2.2. Animals and Ethics

We used 170 male Wistar rats, which were purchased in Stolbovaya breeding nursery (Scientific Center for Biomedical Technologies, FMBA, urban-type settlement Stolbovaya, the Moscow region, Russia). The experiment was performed following the Order of the Ministry of Health of the Russian Federation number 199n dated 1 April 2016 “On approval of the rules of good laboratory practice”. The design of the experiment was approved by the Ethics Committee of Federal Research Centre of Nutrition and Biotechnology (protocol number 2 of 9 September 2013). The rats were housed in groups of 3 animals in the polycarbonate cages under 12/12 h illumination conditions and a temperature of 22 ± 1 °C; diet and water were provided *ad libitum*.

### 2.3. Subacute 92-Day Experiment

**Figure 1:**
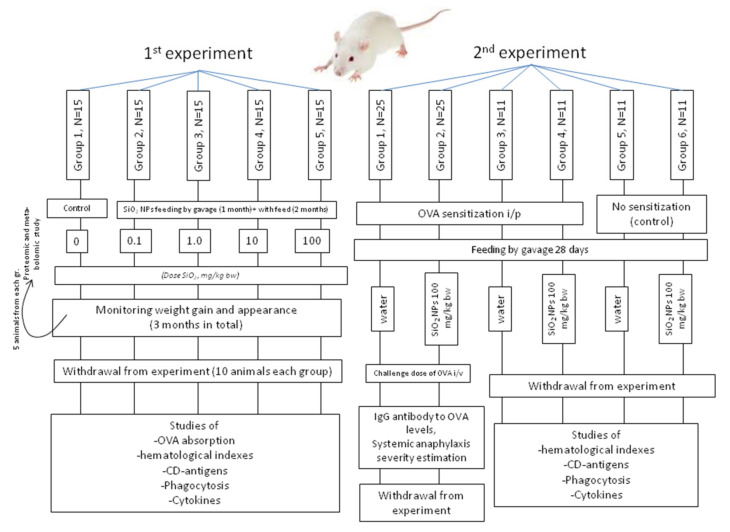
The scheme of the 1st and the 2nd experiments’ designs.

In the first experiment (Figure 1), five groups of animals were formed with 15 rats of the same age (about 7 weeks) in each with an initial average bw of 80 g. During the study, all animals received a balanced semisynthetic diet identical in chemical composition to AIN93 [13]. Animals of group 1 (control) received deionized water which was used as a carrier for NM. Rats in the groups from 2 to 5 received nanostructured SiO_2_ in the form of a suspension in deionized water in the doses 0.1, 1.0, 10, and 100 mg/kg bw based on dry silica, respectively. Before the introduction to animals, the suspension was sonicated as described in Section 2.1. During the first 30 days of the experiment, the NM was administered by gavage, and during the subsequent 62 days, SiO_2_ suspension was added to animal feed; the SiO_2_ doses were calculated based on the consumption of the diet. Throughout the experiment, the rats were weighed daily on an electronic balance with an accuracy of ±1 g; morbidity, mortality, appearance, activity, behavior features, and state of the coat were observed.

Animals were euthanized on day 93 by exsanguination from the inferior vena cava under ether anesthesia. Three hours before the end of the experiment, 8 or 9 rats from each group received by gavage chicken egg ovalbumin (OVA) dissolved in 0.15 M NaCl at a dose of 3 g/kg bw per protein. The weights of the internal organs (liver, kidney, spleen, heart, testis, thymus, lung, adrenal gland) were determined by weighing on an electronic balance with an accuracy of ±0.01 g. Blood was collected in anticoagulant (0.01% by weight of tripotassium ethylene diamine tetraacetate EDTA) and in a dry sterile tube for serum separation.

The intestinal barrier permeability for OVA macromolecules was evaluated by its concentration in the serum, determined by solid phase “sandwich” enzyme-linked immunoassay (ELISA), according to [14], with minor modifications. The magnitude of absorbed OVA amounts in percent of the administered dose was calculated in the whole blood flow based on mean blood mass in the amount of 6% of body weight according to reference data [15] and hematocrit of 44%.

### 2.4. Twenty-Eight-Day Systemic Anaphylaxis Model Experiment

In the second experiment (Figure 1), 95 male Wistar rats with an initial average body weight of 160 g were divided into six groups of 25 (group 1), 25 (group 2), and 11 (groups 3–6) animals. During the experiment, the animals of all groups received a standard diet prepared in the vivarium of the Federal Research Centre of Nutrition and Biotechnology based on natural foods, balanced on macro- and micronutrients. Rats of 1st, 3rd, and 5th groups received by gavage deionized water in the amount of 10 mL/kg bw. Rats of 2nd, 4th, and 6th groups daily received by gavage SiO_2_ NPs at a dose of 100 mg/kg bw as a sonicated aqueous dispersion containing 1% of SiO_2_ NPs by weight. On the 1st, 3rd, and 5th day of the experiment, rats from 1–4 groups were immunized intraperitoneally by a dose of 100 µg of 5-fold recrystallized OVA adsorbed on 10 mg of freshly precipitated aluminum hydroxide as described in [16]. On day 21, the rats of these groups were injected with an additional 10 µg of OVA under the same conditions to induce a secondary immune response. On the 29th day, 0.5 mL of blood was taken from each of the tail veins of rats from groups 1 and 2 for the antibody determination; then 6 mg/kg bw OVA in sterile pyrogen-free 0.15 M NaCl was injected intravenously as a challenge dose for induction of systemic anaphylaxis. The protein solution was pre-filtered through a membrane filter with pores of 0.22 µm. Development the symptoms of an active anaphylactic reaction (AAR) was observed within 24 h after administration of the challenge dose; the severity of the reaction was evaluated in accordance with the scale of “0” being no reaction; “+”—chills, shortness of breath, itching; “++”—weakness, ataxia, cyanosis of the extremities; “+++”—convulsions, paralysis; “++++”—death. Surviving animals of groups 1 and 2 were taken out of the experiment by a lethal dose of CO_2_ inhalation. Rats of the 5th and 6th groups were not immunized and served as controls to groups number 3 and 4 respectively.

Rats in groups 3–6 were taken out of the experiment by exsanguination from the inferior vena cava under deep ether anesthesia. Blood was collected in anticoagulant (0.01% by weight of tripotassium EDTA) for determination of hematological and immune indices, and in a sterile dry test tube for the determination of serum cytokines.

### 2.5. Hematological and Immunological Parameters

Hematological indices were studied on hematology analyzer “Coulter AC TTM 5 diff OV” (company “Beckman Coulter”, Indianapolis, IN, USA) with a standard set of reagents (manufactured by “Beckman Coulter”, Villepinte, France). The defined parameters included the number of erythrocytes, leukocytes, hemoglobin concentration, hematocrit, mean cellular volume, mean corpuscular hemoglobin content, mean cellular hemoglobin concentration, platelet count, mean platelet volume, the relative number of platelets in the whole blood, and white blood cells (WBC) population composition.

Expression of antigens CD45RA, CD3, CD4, CD8, and CD161a on peripheral blood lymphocytes was determined by direct immunofluorescence staining of whole blood with a panel of monoclonal antibodies conjugated with fluorescent dyes, and lysing/fixing reagent kit (manufactured by “Beckman Coulter”, Indianapolis, IN, USA). Lysis/fixation and staining of cells were performed according to the manufacturer’s instructions. Analysis of the stained cells was performed on a flow cytometer “FC-500”, manufactured by “Beckman Coulter”, Indianapolis, IN, (USA) using the software “Cytomics CXP Software” (manufactured by “Beckman Coulter”, Fullerton, CA, USA). Lymphocyte populations were isolated by gating on parameters small angle (FS) and side (SS) of light scattering. Next was the gating of CD3+ lymphocyte populations via fluorescence channels FL1 and SS Lin. Results were recorded on a two-parameter histogram distribution of CD3+ (from gate B) using monoclonal antibodies against CD4 and CD8, detected in fluorescence channel FL5 and FL4, respectively. Similarly, in a separate test the expressions of CD45RA and CD161a were determined. The total contents of CD45RA+ (B-lymphocytes), CD3+ (T-lymphocytes), and CD161a+ (natural killer) were expressed in percentages of the total number of lymphocytes analyzed (at least 104 events per animal). The contents of CD3+CD4+ (T-helper cells) and CD3+CD8+ (cytotoxic T) were determined as percentages of their share in the total number of CD3+ cells. The dimensionless immunoregulatory index (IRI) was calculated, which was the ratio of CD4+/CD8+ cells.

Study of the phagocytic activity of polymorphonuclear leukocytes in peripheral blood was performed by a standard method using a reagent kit Phagocytosis Assay Kit (IgG FITC), manufactured by “Cayman Chemical Company”, Ann Arbor, MI, (USA) according to the instructions attached. The object of phagocytosis was latex particles opsonized IgG FITC. The cell suspension was incubated with the addition of latex to the whole animal blood for 30 min in two test tubes. Samples are analyzed on a flow cytometer using the program Cytomics CXP Software. Populations of neutrophils were isolated by gating on parameter FS, and SS of light scattering was evaluated by the number of cells via phagocytosed value fluorescence FITC. The analysis was performed twice: in the presence and absence of a phagocytosis stimulator—phorbol-12-myristate-13-acetate (PMA), whereupon a stimulation index was calculated for each blood sample studied.

Determination of cytokines IL-6, IL-10, and TNF-α in the serum of rats was performed by ELISA using commercial kits “Bioscience”, produced by “Bender Med-Systems GmbH” (Wien, Austria), following the manufacturer’s recommendations. The intensity of humoral immune response to OVA in rats in groups 1 and 2 (second experiment) was evaluated by circulating specific IgG antibodies level using indirect ELISA on polystyrene. Antibody response was analyzed in terms of the level of antibodies in serum (mg/cm^3^), the level of the decimal logarithm, and the absorbance value in ELISA. Optical density was measured on an automatic immunoassay plate photometer “EFOS 9605” (Moscow, Russia).

### 2.6. Statistics

Statistical analysis was performed using SPSS 20.0 package (SPSS Inc., Chicago, IL, USA) according to the paired Student’s *t*-test, nonparametric Mann–Whitney *U*-test, χ-square criterion, criterion *U* Fisher’s angular transformation, and ANOVA. Differences were considered significant at a level of *p* < 0.05.

## 3. Results

### 3.1. SiO_2_ Characterization

**Figure 2:**
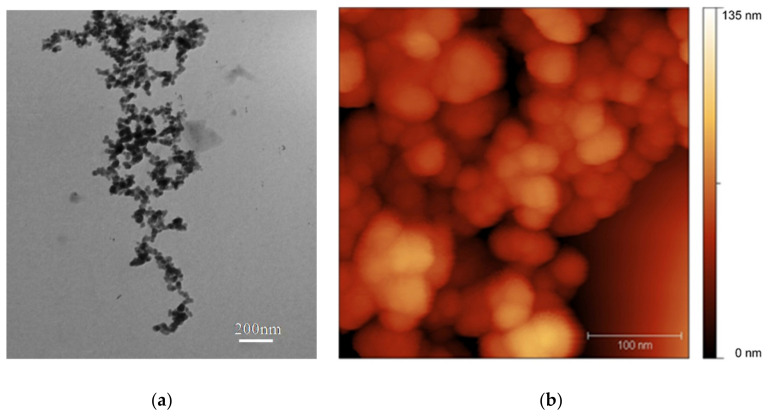
Representative images of SiO_2_ particles obtained by transmission electron microscopy (**a**) and atomic force microscopy (**b**).

A TEM study of SiO_2_ suspension with a concentration of 1 µg/cm^3^ showed that particles on the grid were distributed mainly in the form of large loose aggregates consisting of primary particles with diameters less than 100 nm. AFM showed the presence of NPs, which were mainly in the aggregated state. NPs aggregates were present in all scans with a scanned area of 20 × 20 µm^2^; the sizes of aggregates varied, reaching a maximum value of up to 2 µm (in one of the measurements). It was found that the structural element for all aggregates was a particle with dimensions less than 100 nm. The population of NPs in the composition of aggregates consisted of particles of rounded or nearly spherical morphology with a diameter ranging from 20 to 60 nm. The representative TEM and AFM images of the studied samples of SiO_2_ are presented in Figure 2a,b.

**Figure 3:**
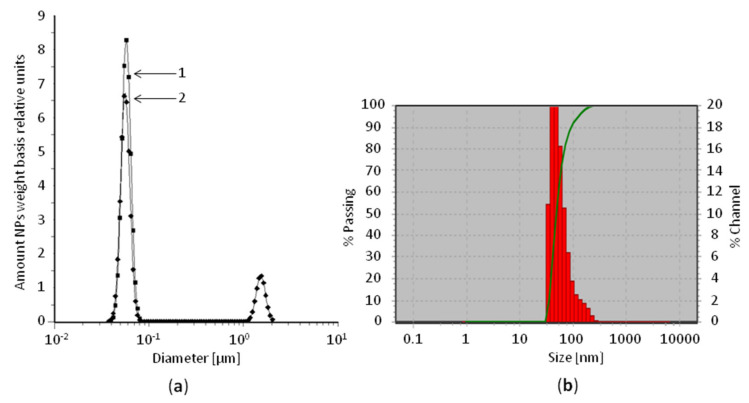
Results of SiO_2_ particle size measurement by (**a**) spectroacoustic study (1—after sonication; 2—before sonication) and (**b**) dynamic light scattering (sonicated). In (**b**) x-axis: particle size, nm. Y-axis: left—the fraction of particles with a diameter at least this, % (line); right—the fraction of particles in the size range, % (histogram).

A spectroacoustic study of an aqueous suspension of SiO_2_ with a concentration of 5% by weight, treated or untreated with ultrasound, revealed a bimodal particle size distribution with a predominance of NPs with an average size of 20–40 nm. Particle size analysis of the sample at a concentration of 1% by weight using dynamic light scattering showed that for the predominant fraction of NPs in the preparation: number average hydrodynamic diameter was 56.6 ± 32.1 nm; 90th size percentile—91.7 nm (Figure 3). The content of particle fraction with a diameter greater than 100 nm after sonication did not exceed 10% of the total number of particles.

Thus, based on the determination of SiO_2_ particle size and shape by four independent methods, it was found that the sample was presented by NM.

### 3.2. Subacute 92-Day Experiment on Non-Immunized Animals

#### 3.2.1. Condition and Growth of Animals during the Experiment

**Figure 4:**
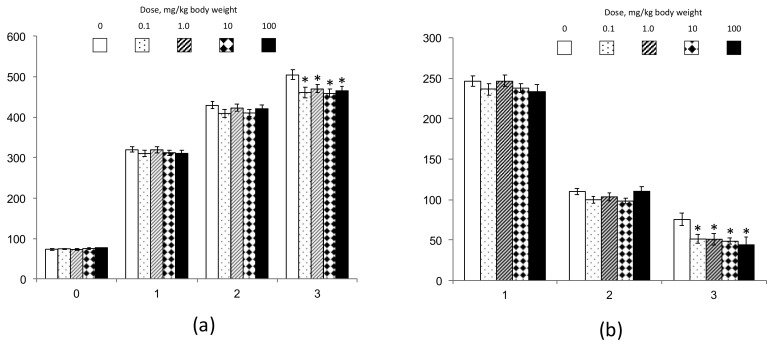
Average body weight (**a**) and monthly weight gain (**b**) of rats in groups 1–5 during the experiment. The x-axis—time, months; y-axis—the average body weight (**a**) or the average increase of body weight (**b**); *—difference with group 1 is significant, *p* < 0.05, Mann–Whitney *U*.

During the first month of intragastric administration of SiO_2_ suspension to animals, the death of one rat occurred in group 4, and three occurred in group 5. The autopsy of dead animals showed the signs of bilateral pneumonia, as might be expected due to accidental aspiration of NM’s traces when administering by gavage of a suspension containing a high concentration of NPs. The presence of inhalation toxicity in SiO_2_ NPs is known from the data of [2,3]. Furthermore, one rat died in group 3 in the third month of the experiment. Other animals of all experimental groups had normal appearances regarding the coat, mucousal surfaces, locomotor activity, behavior, and stool, and did not differ in this respect from the control group. Determination of average monthly increases of bw as follows from data presented in Figure 4 showed that during the first and second months of the experiment, the animals of all groups gained weight at almost the same rate (*p* > 0.1, ANOVA). However, after the third month, there was a slight (not more than 15%) but significant delay in weight gain in all experimental groups compared to the control. This effect was not dose-dependent and was probably associated in aged animals with a reduction of fat mass gain, which cannot be interpreted as a sign of unfavorable (toxic) activities of SiO_2_ administration.

#### 3.2.2. Weights of Internal Organs

The data presented in Table 1 show the relative weights of internal organs expressed as percentages of body weight. No changes in the weight of the majority of the internal organs related to SiO_2_ dose were observed. The exception was the adrenal glands, whose mass was significantly (*p* < 0.05) increased in groups 3 and 4 compared with group 1, a maximum of 32%. In group 5, receiving the highest dose of NM, this effect was not displayed, so it is not dose-dependent.

#### 3.2.3. The Intestinal Barrier Permeability

**Figure 5:**
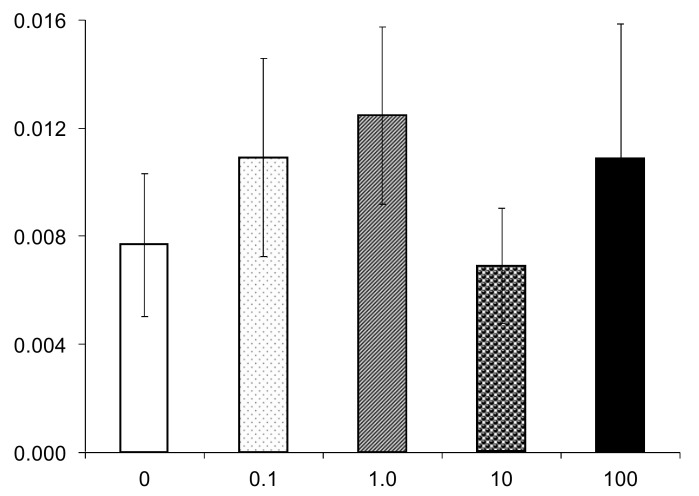
Data characterizing the permeability of the intestinal barrier for macromolecules of protein (OVA) in rats of groups 1–5. X-axis—dose of SiO_2_ NPs, mg/kg bw. Y-axis—absorption of OVA macromolecules into the blood, percentage of the administered dose × 10^3^, *M* ± s.e.m. Data distribution in group is homogeneous (ANOVA *F* = 0.504; *p* > 0.1). Paired differences between groups are insignificant, *p* > 0.1, Mann–Whitney test.

Orally administered NPs of some species may have an irritating effect and induce inflammation in the wall of the small intestine. Given this, it was of interest to find out whether nanostructured SiO_2_ is capable of affecting the permeability of the intestinal barrier for protein macromolecules. The data presented in Figure 5 show that no significant changes were observed in the absorption of macromolecular proteins into the blood in animals that received NPs for three months at a dose of up to 100 mg/kg of bw 

#### 3.2.4. Hematological Indices

The results of hematological parameter determination in animals of groups 1–5 are shown in Table 2 and Table 3. As follows from the data, rats of fourth group had a significant (*p* < 0.05) decrease in the number of red blood cells (5%), which was accompanied by significant (*p* < 0.05) increases in the average volume of red blood cells and hemoglobin content. A small (5%) but significant increase in the mean corpuscular volume of erythrocytes was also observed in the animals of group 5. Other parameters had no significant changes from the data from the remaining experimental groups of animals. There were no changes in the parameters characterizing the state of platelets in animals (*p* > 0.05 for all indicators; data not shown). Thus, even in the highest dose, the magnitude and direction of identified changes in the state of erythrocytes and platelets of animals do not indicate any adverse impact of nanostructured SiO_2_ on blood. Indicators of blood leukocytes (Table 3) did not differ in animals of groups 2–5 from the control, except for a significant (*p* ≤ 0.05) and quite pronounced (33%) decrease in the total number of leukocytes in rats of group 5.

#### 3.2.5. Immunological Parameters in Non-Immunized Animals

**Figure 6:**
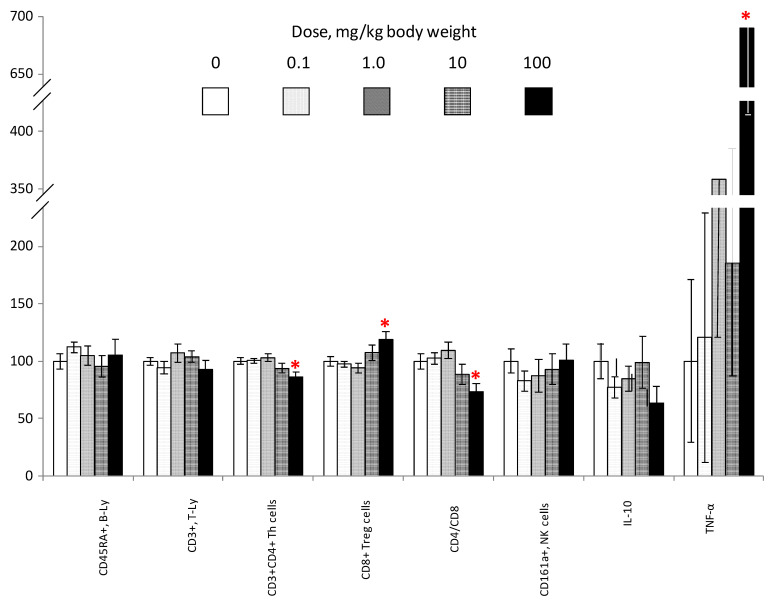
Key indicators of the cellular immunity in rats of 1–5 groups. X-axis—name of indicators, number of samples; y-axis—the values in percent of the control group (group 1), *M* ± s.e.m. *—Difference with group 1 is significant, Student’s *T* and Mann–Whitney *U*.

Although the relative number of lymphocytes (Ly) in the total population of leukocytes shows an only slight (7%) and nonsignificant decrease in animals of group 5, the qualitative composition of Ly, as can be seen from the data presented in Figure 6, was substantially modified. Particularly, the proportion of Th cells (13%) was significantly reduced, and Tregs were more common (19%); all of that led to the decrease (27%) in the immunoregulatory index. In combination with the general decline in the number of identified leukocytes, this indicates a significant weakening of the T-cell immunity in the 5th group receiving the highest doses of SiO_2_ NPs. This observation is supported by data on the occurrence of an imbalance in levels of the main group of pro- and anti-inflammatory cytokines, particularly TNF-α, whose content was significantly increased by an average of 590%, and IL-10, the content of which exhibited a pronounced (36%) tendency toward reduction. It can be assumed that the detected decrease in Th cells extends mainly to the Th1 population. IL-6 levels in the blood of animals in all groups remained below the detection limit of the method (data not shown). Thus, the NOAEL level for nanostructured SiO_2_ in terms of hematological and immunological indices was within the range of 10–100 mg/kg body weight.

### 3.3. Twenty-Eight-Day Experiment on Immunized Animals

It is also of interest to establish the effect of oral administration of nanostructured silica on the performance of the immune system in animals under the conditions of the food allergen immunization model, reproduced with OVA immunization. The period selection of NM administration to animals (28 days) was due to the time of development of allergic immunization in the applied standard model of systemic anaphylaxis [14]. During the 28 days of SiO_2_ administration, one rat died in the second group and one in the sixth group. The autopsy showed signs of bilateral pneumonia, which could have been due to accidental aspiration of the NP suspension in the respiratory tract. The remaining animals had normal appearances, activity, and stool. As can be seen from the data presented in Table 4, the average body weight gains of rats in the first and second groups at the end of the experiment were not significantly different. The data on the severity of the systemic anaphylaxis reaction (Table 4) indicate that in animals that received SiO_2_ NPs at a dose of 100 mg/kg bw per day for a month, an increase in the severity of the allergic reaction was observed, which, however, remained statistically insignificant (*p* > 0.05). The same can be noted in the level of IgG antibodies to OVA, expressed by three different parameters (Table 4). The distributions of animals in groups 1 and 2 by the severity of the anaphylaxis reaction, expressed in points, differed statistically insignificantly (*p* > 0.05, chi-square). Thus, the effect of enhancing immunization by food allergen OVA was weak (marginally) in animals exposed to SiO_2_ NPs at a dose of 100 mg/kg bw; therefore, this level can be considered as the upper limit of NOAEL.

Average indices characterizing the state of erythrocytes in rats of groups 3–6 only slightly (3–5%) differed between groups (data not shown). There were slight but significant (*p* < 0.05, ANOVA) reductions in the concentration of hemoglobin (4%) and the erythrocyte count (3%) in the blood of immunized animals (groups 3 and 4) when compared to non-immunized (groups 5 and 6). No influence of NM on the states of erythrocytes and platelets was found in immunized or non-immunized animals. The study of leukocyte counts showed that immunization and monthly administration of NM did not have any impact on these indicators, except for a significant (*p* < 0.05) increase in the number of total Ly by 8% due to immunization against the introduction of SiO_2_ NPs. It can be assumed from Table 5 that the reason for this increase was probably growth in the number of B cells, the proportion of which in the total pool in immunized animals treated with NPs increased. In the absence of NPs treatment, a similar effect was not observed.

When assessing the parameters of the cellular immune system of animals (Table 5), it can be concluded that both 3-month and 1-month administration of SiO_2_ NPs at a dose of 100 mg/kg bw in non-immunized animals leads to a significant reduction of T-helper cells, increasing the number of cytotoxic T-Ly and reducing the CD4/CD8 ratio. However, these changes are “fuzzy” against the background of immunization and acquire the character of a statistically insignificant trend. Thus, strengthening of SiO_2_ immunotoxic properties in the conditions of food protein immunization was at least not observed. Noteworthy is the significant (*p* < 0.05) and a very pronounced (75%) increase in the number of natural killer cells (CD161a+) as a result of exposure to NPs in non-immunized animals. Against the background of immunization, this effect apparently does not occur. However, these changes can be considered as the activation of nonspecific cellular immunity under the influence of NPs, which cannot be unambiguously interpreted as negative (harmful). Therefore, sufficient reason is absent to reduce the range of NOAEL in the condition of immunization.

Basal indices of the activity of phagocytosis of neutrophilic leukocytes in rats of groups 3–6 did not differ (data not shown). The stimulation index of phagocytosis by the action of PMA showed a tendency to decrease in group 6 under SiO_2_ action in the absence of immunization; the difference with 5th group, however, was insignificant (*p* > 0.05). When comparing similar groups of immunized animals (third and fourth), said effect was apparently not noticeable. Factor analysis showed the absence of any influence on phagocytosis both under immunization and under NM consumption.

Analysis of cytokine levels in the sera of animals showed that there is a tendency to reduce the IL-10 concentration upon administration of SiO_2_ NPs in immunized rats (41%), and one toward an increase in the TNF-α level in unimmunized rats (28%, data not shown). However, these differences were not significant (*p* > 0.05). It can be assumed that the 28-day period of NM administration was apparently insufficient to develop pronounced effects on cytokine production, which were identified in a three-month experiment.

Thus, the 28-day administration of nanostructured SiO_2_ at a dose of 100 mg/kg bw, which was the largest studied, does not lead to a significant increase in allergic sensitivity against a food allergen. On the other hand, potentially unfavorable changes in the immune system, observed at the highest dose of NM, particularly the balance of CD4+ and CD8+ cells, are at least not increased on the background of immunization. Thus, the data of the immunological and allergy study in immunized animals did not give reasons for the revision of the NOAEL level, established in experiments on non-immunized animals.

## 4. Discussion

In this work, the study of the effect of nanostructured SiO_2_ administered orally to rats on some biological indicators of toxicity, including the parameters of the cellular immune system, was carried out in the dose range from 1 to 100 mg/kg of bw in a subacute 92-day experiment. According to a previous evaluation [6], the daily load of SiO_2_ NPs from food on a person can reach 1.8 mg/kg of bw, which is close to the minimum daily dose used in our present study. The maximum 100-fold aggravated dose of NM was at least 50 times lower than the corresponding LD_50_, which was estimated at 5000 mg/kg bw. Further aggravation of SiO_2_ dose in this experiment was not possible, as this greatly increased the risk of the animals’ death due to the aspiration of the suspension traces during the first month of administration.

The data obtained in hematological and immunological studies showed that the administration to animals of nanostructured SiO_2_ at a dose of at least 100 mg/kg bw led to a noticeable shift in the parameters of the immune system, which may indicate the manifestation of a certain toxic effect. This result is consistent with many published data on the toxicity of silica NPs.

According to in vitro studies, amorphous SiO_2_ NPs have various toxic properties. The adverse effect of NPs may be based on the catalytic generation of free radical compounds found in the cell-free system [17], in cultured keratinocytes [18], and human alveolar epithelial cells [19]. Cytotoxic effects of SiO_2_ NPs for the EAHY926 cell line were observed in a study [20]. Under the same conditions, submicron particles (100–330 nm) were not toxic. In the culture of mouse embryonic stem cells, amorphous SiO_2_ NPs 10 and 30 (but not 80) nm in diameter suppressed differentiation into normal cardiomyocytes [21]. Apoptosis and changes in the expression of p53, Bax, and Bcl-2 were found in normal liver cells of the L-02 line under the action of 21 nm SiO_2_ NPs [22]. The presence of various cytotoxic properties in SiO_2_ NPs was also revealed in the works [23,24,25]. There is also some evidence on the organotoxic action of silica NPs in vivo. Hence, a cardiotoxic effect was observed after the parenteral administration of silica NPs to zebrafish embryos, which manifested in inflammation of the heart muscle, mediated by suppression of the transcriptional activity of the genes ATPases, calcium channels, and troponin C [26].

According to a number of studies, NPs of different types [27,28,29,30,31] can be absorbed in the digestive tract, thereby entering the circulatory and lymphatic systems. The quantitative assessment of the absorption and bioavailability of SiO_2_ NPs is a difficult task due to the lack of methods for detecting these NPs in complex matrixes [32]. In [6], for the first time, indirect evidence was obtained for the accumulation of silicon in the liver after 84-day intragastric administration of SiO_2_ NPs to rats. Unfortunately, the authors used the ICP-MS method, which made it impossible to estimate the size of accumulating NPs. In addition, high background levels of silicon in animal tissues made it impossible to rigorously quantify this indicator. A number of in vivo studies have shown that nanostructured amorphous SiO_2_ exhibits inhalation toxicity [2,3]. However, the study of the effects of SiO_2_ NPs after oral administration has led to contradictive results. Intragastric administration of nanostructured amorphous SiO_2_ to rats at a dose of 170–1500 mg/kg bw within 90 days did not cause pronounced toxic effects, which, apparently, was studied by a complex of biochemical, physiological, hematological, and allergological parameters [7]. After the exposure of zebrafish eggs to silica NPs, no subsequent occurrence of anomalies in the development of embryos, or signs of cardio- and hepatotoxicity was revealed, although there were some features in behavioral reactions [33]. Feeding female quails with SiO_2_ nanoparticles for 10 weeks at a dose of up to 4000 mg per kg of feed did not lead to significant changes in blood plasma biochemical parameters and histopathological changes in the liver, but caused a decrease in the weight of the yolks of eggs and an increase in the thickness of their shells [34]. Based on the data listed, it may be concluded that the oral toxicity of silica nanoparticles is relatively low. At the same time, in works [3,4], mice in an acute experiment were injected intragastrically with “monodisperse” SiO_2_ NPs obtained by the method of the liquid crystal templating using cetyltrimethylammonium bromide as a template. The SiO_2_ particles used in these studies were elliptical in size of approximately (50~70) × (20~30) nm. In the groups receiving NPs, on days 3 and 4, the death of animals was observed at an LD_50_ level of 4600 mg/kg bw. At a dose of SiO_2_ NPs higher than 0.2 × LD_50_, the damage to blood cells was observed, and at a dose of 0.3 × LD_50_, morphological changes in the circulatory, lymphoid, and macrophage systems, and degenerative changes in the liver and kidneys were seen. The studied SiO_2_ NPs were characterized by significant cumulativeness (cumulative index 0.45).

The authors of the article [6] studied the subacute 84-day toxicity of two types of nanostructured silica when administered orally to rats at very high doses (100–2500 mg/kg bw daily). Dose-dependent increasing fibrosis in the liver and the expression of genes responsible for this process have been noted. According to these alterations, the authors estimated the lowest observed adverse effect level (LOAEL) for SiO_2_ NPs at 2500 mg/kg bw. However, the performance of the immune system in this study has not been investigated. It can be assumed that the main reason for the discrepancy in results [6,7] and [3,4], was the difference in the properties of the SiO_2_ preparations used. It should be noted that all types of nanostructured SiO_2_ studied in [2,3,4,5,6,7] are not used in consumer goods, including food. The study of subacute oral toxicity of NPs with a primary particle size of 5–30 nm showed the absence of noticeable signs of toxic effects when NM was administered for three months at a dose of up to 100 mg/kg bw daily by integral indicators, including weight gain, and permeability of the intestinal barrier to macromolecules. The identified single effects (adrenal hypertrophy in animals of the third and fourth groups) were relatively small in magnitude and did not show a clear dose-dependence. The main “target” of orally administered SiO_2_ NPs is the immune system—specifically, T-cell immunity. According to [11], it is assumed that the immunotoxic effect of silica NPs is mediated by the formation of active oxygen substances (ROS) that affect the MAPK pathway, the activation of NLRP3 inflammasomes, and Toll-like receptors with the subsequent induction of autophagy and apoptosis of immune cells. As a result, the number of T-helpers decreases, cytotoxic T-lymphocytes increase, the immunoregulatory index decreases, and the levels of circulating TNF-α increase. As is known, TNF-α is a proinflammatory cytokine synthesized by activated macrophages at the early stages of the immune response. It exhibits cytotoxicity and activation of catabolism, and provides many immunoregulatory effects [35,36]. There is evidence, in particular, of its ability to suppress the activity of helper T cell-mediated immunity, which plays a role in the pathogenesis of HIV infection [37]. The revealed increased production of TNF-α under the influence of SiO_2_ NPs (100 mg/kg bw) can be considered as one of the indicators of immunotoxic action together with a decrease in the level of T-helpers and the magnitude of the immunoregulatory index.

One of the possible consequences of the immunotoxic effect of SiO_2_ NPs may be an ability to enhance allergic reactions to extraneous antigens, which was found in [11] in the intranasal route of these NPs’ administration. However, our attempt to reveal an increase in the allergic reaction to OVA on the model of systemic anaphylaxis under the influence of intragastric intake of SiO_2_ NPs was essentially unsuccessful, showing only a marginal effect which was manifested at the level of a statistically insignificant trend. Thus, it can be assumed that oral intake of nanostructured silica does not pose any significant risk of increased allergic reactions.

When discussing the possible mechanisms of SiO_2_ NPs’ action on immune cells in vivo, it should be noted that there are data in the literature on the adverse effects of various NM on immunological and hematological parameters. Among them are increased production of proinflammatory cytokines [38], platelet aggregation [39], and hemolysis [40]. However, these results were obtained in tests in vitro, where the effective dose of NPs can be significantly increased compared to in vivo conditions. When zebrafish embryos were injected into the bloodstream with SiO_2_ NPs, they reduced the blood flow rate, caused hypercoagulability, increased erythrocyte aggregation, and induced endothelial inflammation mediated by the JAK1/TF signaling pathway [8]. Full-transcriptome analysis in zebrafish embryos subjected to silica NP action showed increased expression of 1107 genes and suppression of 1408 genes, including the genes involved in the regulation of the immune response; and the production of cytokines, such as genes of the JAK-STAT signaling pathway [41]. Intraperitoneal injection of SiO_2_ NPs into rats showed shifts in the function of peritoneal macrophages; increased production of signaling molecules such as IL-1β, TNF-α, and NO; and increased gene expression of IL-1β, TNF-α, nitric oxide synthase, and cyclooxygenase-2 [42]. According to [2], inhalation of SiO_2_ NPs in mice caused pulmonary neutrophilia, accompanied by increased expression of TNF-α and neutrophil-attracting chemokine CXCL1 in the lung tissue. SiO_2_ NPs were also able to enhance the allergic immunization with OVA during intranasal [11] and inhalation [43] treatment of mice and rats. In parenteral administration, some oxide NPs activate the kallikrein-kinin system [44] or are histamine liberators [45]. The pro-allergenic effect of inhaled NPs—in particular those produced on an industrial scale—must be taken into account when establishing hygienic standards for occupational exposure, while for NPs contained in food and drinking water, these risks seem to be considerably lower.

**Figure 7:**
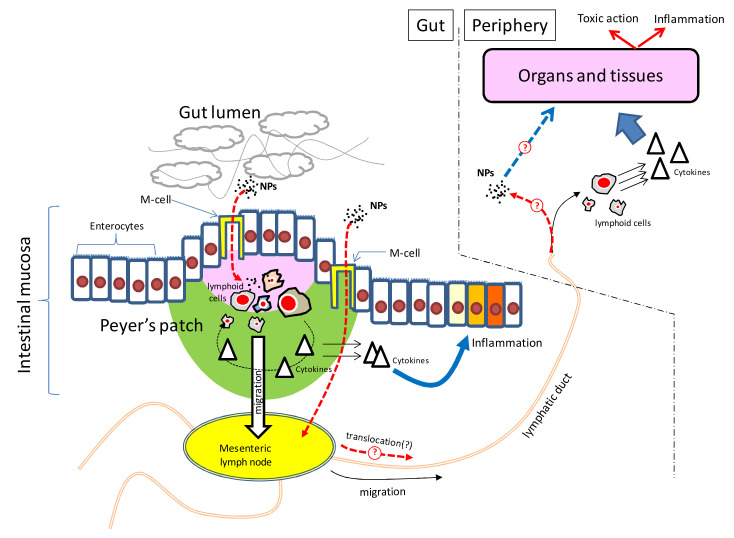
Hypothetical mechanism of SiO_2_ NPs’ immunotoxic action at an oral way of administration.

The data of the publications discussed above leave open the question of how the immunotoxic effect of silica NPs can manifest itself after the oral administration, given the apparently very low systemic bioavailability of these NPs from the gastrointestinal tract [6]. To explain the reactions identified in our investigation, it is useful to take into account the study of Zaitseva et al. [46], which under conditions close or identical to our work, showed a pronounced local immune response in the intestinal mucosa in rats fed with silica NPs, which manifested as massive leukocyte infiltration and the development of local inflammation. It can be assumed that as a result of the local irritating effect of silica NPs, gastrointestinal lymphoid tissue (GALT) is activated, which is accompanied by the release of circulating pro-inflammatory cytokines, which have a systemic immunotoxic effect, similarly to how it is observed for various inflammatory bowel diseases [47]. It should be noted that this hypothetical mechanism of SiO_2_ NPs immunotoxic action does not necessarily require their translocation into internal organs in significant quantities (Figure 7).

## 5. Conclusions

Thus, SiO_2_ NPs in subacute oral administration to rats lasting up to three months showed effects on the cellular components of the immune system that were, firstly, clearly dose-dependent, i.e., manifesting to the greatest extent at the highest dose; and secondly, indicated by their direction (in particular, reduced the number of T-helper cells and increased production of pro-inflammatory TNF-α) the presence of the toxic action. A lack of significant shifts of these parameters in the group of animals treated with studied NM at a dose of 10 mg/kg bw allowed us to conclude that NOAEL for nanostructured SiO_2_ ranges up to 100 mg/kg bw per day in terms of its daily intakes for 1–3 months. This result indicates that the use of nanostructured silicon dioxide as a food additive, and an ingredient for cosmetic products (such as toothpaste) and pharmaceuticals, must be the subject of hygienic regulations.

## Figures and Tables

**Table 1:** Relative weights of rats’ internal organs in groups 1–5 on the 93rd day of the experiment.

Groups	SiO_2_ Dose, mg/kg Body Weight	Number of Animals ^1^	Mass of Internals, % of Body Weight, *M* ± s.e.m.							
			Liver	Kidneys	Spleen	Testis	Heart	Thymus	Lungs	Adrenals
1	0	9	2.77 ± 0.06	0.628 ± 0.017	0.347 ± 0.018	0.737 ± 0.029	0.284 ± 0.009	0.139 ± 0.008	0.476 ± 0.013	0.022 ± 0.001
2	0.1	10	2.67 ± 0.16	0.631 ± 0.036	0.321 ± 0.018	0.733 ± 0.026	0.274 ± 0.013	0.131 ± 0.012	0.493 ± 0.027	0.024 ± 0.003
3	1.0	9	2.65 ± 0.06	0.643 ± 0.018	0.347 ± 0.024	0.770 ± 0.016	0.266 ± 0.005	0.142 ± 0.006	0.501 ± 0.014	0.028 ± 0.001 *
4	10	9	2.59 ± 0.07	0.629 ± 0.015	0.321 ± 0.007	0.822 ± 0.040	0.268 ± 0.006	0.156 ± 0.006	0.477 ± 0.020	0.029 ± 0.002 *
5	100	7	2.71 ± 0.04	0.639 ± 0.016	0.306 ± 0.015	0.769 ± 0.025	0.280 ± 0.009	0.130 ± 0.011	0.480 ± 0.019	0.027 ± 0.002
Homogeneity of distribution in groups 1–5, ANOVA			*p* > 0.05 *F* = 0.446	*p* > 0.05 *F* = 0.083	*p* > 0.05 *F* = 0.969	*p* > 0.05 *F* = 1.644	*p* > 0.05 *F* = 0.768	*p* > 0.05 *F* = 1.363	*p* > 0.05 *F* = 0.330	*p* > 0.05 *F* = 1.730

^1^ From each group of animals in this experiment, 5 rats were removed from the study for examination of metabolomic and proteomic parameters of the liver; the experimental methodology used therein did not allow including these animals in the data presented in this study. *—Difference with group 1 is significant, *p* < 0.05, Mann–Whitney *U*.

**Table 2:** Erythrocyte indices in groups 1–5 on the 93rd day of the experiment.

Groups	Number of Animals ^1^	Indices, *M* ± s.e.m.					
		Number of Erythrocytes, 10^12^/dm^3^	Hemoglobin Concentration, g/dm^3^	Hematocrit, %	Mean Volume of Erythrocyte, µm^3^	Mean Content of Hemoglobin in Erythrocyte, pg	Hemoglobin Concentration in Cells, g/dm^3^
1	9	8.63 ± 0.17	147.7 ± 2.5	42.73 ± 0.80	49.56 ± 0.87	17.12 ± 0.32	345.8 ± 2.2
2	10	8.33 ± 0.11	145.3 ± 1.5	41.72 ± 0.55	49.90 ± 0.48	17.43 ± 0.12	348.1 ± 2.3
3	9	8.27 ± 0.10	143.8 ± 3.5	41.91 ± 1.10	50.67 ± 0.87	17.33 ± 0.26	342.7 ± 2.0
4	9	8.15 ± 0.09	147.4 ± 1.9	43.01 ± 0.49	52.89 ± 0.54	18.11 ± 0.25	342.8 ± 2.6
5	7	8.43 ± 0.14	150.1 ± 2.4	44.01 ± 0.64	52.29 ± 0.78	17.81 ± 0.30	340.9 ± 1.5
Homogeneity of distribution in groups 1–5, ANOVA, *p* (*F* value)		>0.05 (2.252)	>0.05 (0.922)	>0.05 (1.387)	0.006 (4.198)	>0.05 (2.461)	>0.05 (1.693)
Significance * compared to group 1, *p*	Group 2	>0.05/>0.05	>0.05/>0.05	>0.05/>0.05	>0.05/>0.05	>0.05/>0.05	>0.05/>0.05
	Group 3	>0.05/>0.05	>0.05/>0.05	>0.05/>0.05	>0.05/>0.05	>0.05/>0.05	>0.05/>0.05
	Group 4	0.020/0.031	>0.05/>0.05	>0.05/>0.05	0.005/0.008	0.026/0.027	>0.05/>0.05
	Group 5	>0.05/>0.05	>0.05/>0.05	0.039/>0.05	0.039/0.043	>0.05/>0.05	>0.05/>0.05

^1^—See comment in Table 1; *—Student’s *T* (numerator); Mann–Whitney *U* (denominator).

**Table 3:** Leucocyte counts in groups 1–5 on the 93rd day of the experiment.

Groups	Number of Animals	Indices, *M* ± s.e.m.						
		Total Leucocytes, 10^9^/dm^3^	Neutrophils, %	Eosinophiles, %	Basophiles, %	Lymphocytes, %	Monocytes, %	Immature Cells, %
1	9	16.79 ± 1.18	18.11 ± 1.54	2.30 ± 0.41	0.29 ± 0.04	70.21 ± 2.15	9.00 ± 0.96	0.73 ± 0.09
2	10	15.10 ± 1.55	20.52 ± 1.58	2.69 ± 0.42	0.28 ± 0.05	67.47 ± 1.80	8.79 ± 1.15	0.95 ± 0.12
3	9	15.72 ± 1.38	19.70 ± 1.99	2.56 ± 0.25	0.28 ± 0.03	70.59 ± 1.91	7.54 ± 0.46	0.89 ± 0.14
4	9	16.37 ± 1.48	20.41 ± 1.91	2.63 ± 0.27	0.32 ± 0.07	68.24 ± 2.03	8.20 ± 0.93	0.91 ± 0.13
5	7	11.21 ± 2.01	24.10 ± 2.65	2.47 ± 0.44	0.30 ± 0.02	65.43 ± 2.51	7.51 ± 0.49	0.91 ± 0.18
Homogeneity of distribution in groups 1–5, ANOVA, *p* (*F* value)		>0.05 (1.861)	>0.05 (1.160)	>0.05 (0.180)	>0.05 (0.143)	>0.05 (0.960)	>0.05 (0.580)	>0.05 (0.428)
Significance * compared to group 1, *p*	Group 2	>0.05/>0.05	>0.05/>0.05	>0.05/>0.05	>0.05/>0.05	>0.05/>0.05	>0.05/>0.05	>0.05/>0.05
	Group 3	>0.05/>0.05	>0.05/>0.05	>0.05/>0.05	>0.05/>0.05	>0.05/>0.05	>0.05/>0.05	>0.05/>0.05
	Group 4	>0.05/>0.05	>0.05/>0.05	>0.05/>0.05	>0.05/>0.05	>0.05/>0.05	>0.05/>0.05	>0.05/>0.05
	Group 5	0.025/0.050	>0.05/>0.05	>0.05/>0.05	>0.05/>0.05	>0.05/>0.05	>0.05/>0.05	>0.05/>0.05

*—Student’s *T* (numerator); Mann–Whitney *U* (denominator).

**Table 4:** Comparison of body weight gain, active anaphylactic reaction (AAR) indices, and antibody response in rats of groups 1 and 2 immunized with ovalbumin (OVA).

Indices, Units	Number of Animals		Significance, *p*	Comment
	Group 1 (N = 24) ****	Group 2 (N = 25)		
Body weight, g, *M* ± s.e.m.	295.5 ± 4.4	284.7 ± 4.2	>0.05 *	-
AAR, lethality (4+), %	33.0	48.0	>0.05 **	-
AAR, heavy reactions (3+ and 4+), %	33.0	48.0	>0.05 **	All animals with heavy AAR died
Anaphylactic index (mean score of AAR)	2.29	2.44	>0.05 ***	-
Level of IgG to OVA, mg/cm^3^, *M* ± s.e.m.	5.53 ± 0.90	6.14 ± 0.98	>0.05 * >0.05 ***	-
Level of IgG to OVA, optical density units at 492 nm, *M* ± s.e.m.	1.316 ± 0.043	1.345 ± 0.038	>0.05 * >0.05 ***	-
lg[level of IgG to OVA]	0.580 ± 0.088	0.637 ± 0.081	>0.05 * >0.05 ***	

*—Student’s *T*; **—Criterion *U* Fisher’s angular transformation; ***—Mann–Whitney *U*; **** One animal in group 1 was not challenged (i.v. injection of antigen failed).

**Table 5:** Cellular immunity indices in rat groups 3–6 on the 29th day of the experiment.

Groups	Number of Animals	OVA Immunization +/−	Dose of SiO_2_, mg/kg bw	Indices, *M* ± s.e.m.					
				Number of CD45RA+ Cells (B-Ly), %	Number of CD3+ Cells (T-Ly), %	Number of CD3+CD4+ (T-Helpers), %	Number of CD3+CD8+ Cells (T-Cytotoxic), %	Ratio CD4/CD8	Number of CD161a+ Cells (Natural Killers), %
3	10	+	0	37.5 ± 2.5	47.5 ± 2.6	52.5 ± 4.3	44.85 ± 4.5	1.42 ± 0.29	5.13 ± 0.77
4	10	+	100	36.8 ± 3.2	50.8 ± 3.7	49.4 ± 3.2	48.25 ± 3.2	1.10 ± 0.15	5.18 ± 0.44
5	10	−	0	36.8 ± 3.3	49.9 ± 2.2	54.2 ± 3.3	43.15 ± 3.2	1.38 ± 0.19	4.32 ± 0.69
6	9	−	100	28.7 ± 3.0	56.9 ± 3.0	42.7 ± 3.8	56.35 ± 3.7	0.82 ± 0.12	7.58 ± 0.93
Homogeneity of distribution in groups 1-5, ANOVA, *p*				>0.05	>0.05	>0.05	>0.05	>0.05	0.021
Paired significance, *p* *		gr.3-gr.4		>0.05/>0.05	>0.05/>0.05	>0.05/>0.05	>0.05/>0.05	>0.05/>0.05	>0.05/>0.05
		gr.5-gr.6		>0.05/>0.05	>0.05/>0.05	0.035	0.014	0.026	0.011
		gr.3-gr.5		>0.05/>0.05	>0.05/>0.05	>0.05/>0.05	>0.05/>0.05	>0.05/>0.05	>0.05/>0.05
		gr.4-gr.6		>0.05/>0.05	>0.05/>0.05	>0.05/>0.05	>0.05/>0.05	>0.05/>0.05	0.039/0.037
Factorial analysis, ANOVA, *p*		Factor of NPs		>0.05	>0.05	>0.05	0.034	0.033	0.042
		Factor of immunization		>0.05	>0.05	>0.05	>0.05	>0.05	>0.05

*—Student’s *T* (numerator); Mann-Whitney *U* (denominator).
